# Longitudinal Remote Sleep and Cognitive Research in Older Adults With Mild Cognitive Impairment and Dementia: Prospective Feasibility Cohort Study

**DOI:** 10.2196/72824

**Published:** 2025-05-28

**Authors:** Victoria Grace Gabb, Jonathan Blackman, Hamish Morrison, Haoxuan Li, Adrian Kendrick, Nicholas Turner, Rosemary Greenwood, Bijetri Biswas, Amanda Heslegrave, Elizabeth Coulthard

**Affiliations:** 1 Bristol Medical School Translational Health Sciences University of Bristol Bristol United Kingdom; 2 Neurology Department Southmead Hospital North Bristol NHS Trust Bristol United Kingdom; 3 Mental Health NIHR Bristol Biomedical Research Centre Bristol United Kingdom; 4 Bristol Royal Infirmary University Hospitals Bristol and Weston NHS Trust Bristol United Kingdom; 5 King's College London London United Kingdom; 6 King's College Hospital London United Kingdom; 7 University of the West of England Bristol United Kingdom; 8 Bristol Trials Centre, Bristol Medical School University of Bristol Bristol United Kingdom; 9 Research & Innovation University Hospitals Bristol and Weston NHS Foundation Trust Bristol United Kingdom; 10 York Trials Unit Department of Health Sciences University of York York United Kingdom; 11 School of Electrical, Electronic and Mechanical Engineering University of Bristol Bristol United Kingdom; 12 Institute of Neurology University College London London United Kingdom; 13 UK Dementia Research Institute University College London London United Kingdom

**Keywords:** feasibility, sleep, remote study design, mild cognitive impairment, dementia, electroencephalography, actigraphy, saliva, digital biomarkers

## Abstract

**Background:**

Sleep holds promise as a modifiable risk factor for neurodegenerative diseases and dementia. Clinical trials to modify sleep in people at risk of or in the early stages of dementia are needed. Monitoring natural sleep from home could support pragmatic and decentralized large-scale clinical trials. However, whether longitudinal sleep research can be successfully delivered remotely in this population has not been established yet.

**Objective:**

We investigated the feasibility of remote longitudinal research using wearable devices, web-based cognitive tasks, and a smartphone app to record sleep and cognition in older adults with mild cognitive impairment (MCI) or dementia.

**Methods:**

Older adults with MCI or dementia due to Alzheimer disease or Lewy body disease and cognitively healthy participants completed at-home sleep and circadian monitoring (digital sleep diaries, actigraphy, wearable sleep electroencephalography, and saliva samples) and digital cognitive assessments for 8 weeks. Feasibility outcomes included recruitment, retention, and data completeness.

**Results:**

In total, 41 participants consented (n=10, 24% participants with Alzheimer disease; n=11, 27% participants with Lewy body disease; and n=20, 49% controls). There were predominantly male and White British participants, with a mean age of 70.9 (SD 5.9) years. Retention was very high, with 40 (98%) participants completing 8 weeks of remote monitoring. Data completeness for sleep electroencephalography was 91% and ranged from 79% to 97% for all remote tasks and was overall high across all participant subgroups. In total, 30% (12/40) of participants reported receiving external support with completing study tasks.

**Conclusions:**

High rates of retention, data completeness, and data quality suggested that longitudinal multimodal sleep and cognitive profiling using novel and remote monitoring technology is feasible in older adults with MCI and dementia and healthy older adults, even without study partner support. Remote monitoring should be considered for mechanistic and interventional trials. Careful consideration should be given to how to ensure remote monitoring technologies reduce burden and enhance inclusivity, particularly in communities traditionally underserved by research and those with lower digital literacy.

**International Registered Report Identifier (IRRID):**

RR2-10.2196/52652

## Introduction

### Background

Sleep disturbances such as insomnia, fragmented sleep, daytime sleepiness, and sleep-disordered breathing are common features of Alzheimer disease (AD) and Lewy body disease (LBD) and often appear early in the disease course and before clinical diagnosis [1,2]. Short and fragmented sleep are associated with processes linked to neurodegeneration, including reduced glymphatic clearance of waste [3], increased amyloid beta (Aβ) and tau burden [4], increased neuroinflammation [5], and impaired cardiovascular health [6]. Several small studies of sleep apnea treatment have demonstrated improvement in cognitive outcomes and blood biomarkers of Aβ and tau, suggesting that sleep interventions could improve prognosis in mild cognitive impairment (MCI) and dementia [7-9]. Poor quality or insufficient sleep in midlife increases the risk of all-cause dementia and MCI [10,11], indicating that improving sleep may also protect against dementia. Large-scale clinical studies are needed to enhance our mechanistic understanding of sleep, confirm the most promising therapeutic targets, and monitor the effectiveness and safety profile of sleep interventions [12,13].

However, selecting sleep assessment tools is challenging, particularly in populations with cognitive impairment. Self-report is convenient, inexpensive, and scalable, and has often been used to examine sleep in people with dementia and MCI [14]; however, it is potentially prone to recall bias due to memory deficits or anosognosia. Self-report also correlates poorly with objective sleep measures, particularly in participants with MCI or dementia [15,16] and those with subjectively poor sleep [17], and cannot inform on key components of sleep such as sleep staging or microarchitecture. Polysomnography (PSG) is typically considered the gold standard for sleep measurement, as it provides rich objective sleep data. However, PSG typically requires expert setup, analysis, and a controlled clinic environment, meaning it is expensive, not easily scalable, and therefore typically used for 1 or a few nights [18,19]. PSG setup, especially in an artificial environment, may also not reflect natural sleep [19] as it disrupts usual routines. Longitudinal data collection in sleep research would be beneficial for monitoring clinical trials and disease progression and could account for variation in sleep from external factors such as acute illness or stress, as well as natural intraindividual sleep variation [19].

Wearable devices, smartphone apps, and telemedicine, apart from actigraphy devices, have rarely been used in MCI or dementia research [20], but offer an opportunity to collect objective and subjective data longitudinally in a natural setting [21], often at relatively low cost. Detailed and accurate sleep analysis can now be achieved through wireless technology, including electroencephalography (EEG) headbands and overnight pulse oximetry [21,22]. While actigraphy has been used in dementia research, EEG headbands and pulse oximetry are less well tested, especially in the earlier stages of impairment [23-25]. There is also increasing interest in remote cognitive testing and digital biomarkers for diagnosis and monitoring progression [26]. Despite this, the adoption of digital health technologies into neurology clinical trials, particularly in older people, has been slow [27].

Remote sleep and cognitive data collection has the potential to decentralize clinical trials, making research more convenient for participants and reducing the costs, participant burden, and carbon footprint associated with study visits, while enabling real-time longitudinal data collection of treatment effects. Improved digital access and literacy among older adults [28] and the use of technology among patients living with MCI or dementia to support independent living and for recreation indicate increasing acceptance of technology [29]. However, not all older adults are comfortable using technology, and changes in cognition, sensory processing, and communication might negatively impact the usability and acceptability of novel devices for research purposes in people with cognitive impairment [29,30]. The few studies that have tested wearable devices and digital health technologies for sleep and dementia research in the home have predominantly collected feasibility data for a single device across only a few nights [31] and required support from a study partner or care home staff [32-34]. Before trials invest in digital health technologies and remote study designs, it is important to know whether participants can and are willing to engage and provide data in such studies over a longer period and how much support might be required.

### Objectives

This study aimed to establish the feasibility of predominantly technology-based longitudinal sleep and cognitive assessments from home in older adults with and without MCI or dementia.

## Methods

### Overview

The Remote Evaluation of Sleep to Enhance Understanding of Early Dementia (RESTED) study was a prospective longitudinal observational cohort study involving remote sleep and cognitive monitoring of participants with MCI or dementia and age-matched cognitively healthy controls. The full study protocol has been previously published [35]. The study has been reported in line with the STROBE (Strengthening the Reporting of Observational Studies in Epidemiology) guidelines [36] (Multimedia Appendix 1).

### Ethical Considerations

This study was approved by the Health Research Authority (Yorkshire and the Humber-Bradford Leeds Research Ethics Committee, reference 21/YH/0177). This study was conducted in accordance with the principles of the Declaration of Helsinki. All participants provided written informed consent before engaging in any study activities and were reminded of their right to withdrawal without giving a reason and that withdrawal would not affect their health care. Following consent, data were pseudonymized with participants allocated a participant identifier code to maintain privacy. Participants were not compensated for their involvement in the study; however, travel expenses for research visits outside of usual clinic visits were reimbursed. All researchers engaging with participants were trained in good clinical practice.

### Study Population

The RESTED study recruited community-dwelling adults aged ≥50 years with internet access at home. Participants were recruited to 1 of 3 participant subgroups according to clinical diagnosis meeting standardized diagnostic criteria [37-40]. Participants with MCI or mild dementia due to probable AD were recruited to the AD group, participants with MCI or mild dementia due to probable LBD or Parkinson disease (PD) were recruited to the LBD group, and sex- and age-matched individuals with no known neurodegenerative conditions or cognitive impairment were recruited as controls. Exclusion criteria included advanced dementia, acute or terminal illness, and significant unrelated comorbidities that might interfere with sleep, except for obstructive sleep apnea (OSA) if participants were already undergoing treatment.

Participants were recruited from the city of Bristol, United Kingdom, and the surrounding areas via cognitive and movement disorders clinics and research volunteer databases, including Join Dementia Research. Our original target sample size was 75 participants [41]. Due to the COVID-19 pandemic, the study was delayed, and the budget was partially reallocated to studies that required no patient contact [12,13,42]. Therefore, we revised the target sample size to 40 participants and recruited from a single site (North Bristol NHS Trust).

We did not recruit or require participants to have a study partner. Participants were welcome to invite someone to support them, and study support was recorded.

### Study Procedures

#### Screening

Participants were prescreened for eligibility over the telephone and invited to complete consent and screening in person. Participants who scored less than 11 out of 30 on the Montreal Cognitive Assessment (MoCA) [43] at screening were considered too clinically impaired to participate and were withdrawn.

#### Baseline Assessments

Eligible participants completed baseline questionnaires with a researcher to assess sleep quality (Pittsburgh Sleep Quality Index [PSQI]) [44]; daytime sleepiness (Epworth Sleepiness Scale) [45]; OSA risk (STOP-Bang) [46]; symptoms of depression (Geriatric Depression Scale-15 item) [47]; anxiety (Generalized Anxiety Disorder-7 item) [48]; and apathy (Apathy Evaluation Scale-Self) [49]. Demographic information and medical histories were also recorded.

**Figure 1:**
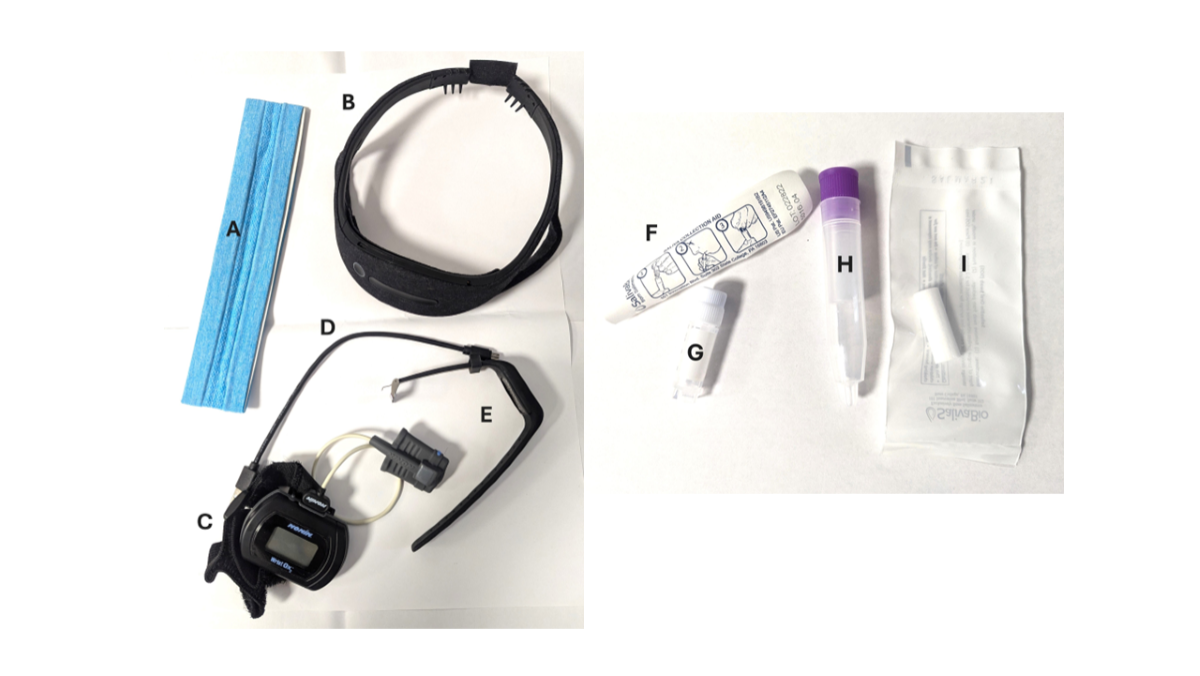
The study kit provided to the Remote Evaluation of Sleep to Enhance Understanding of Early Dementia (RESTED) participants. Participants were additionally required to use a smartphone or tablet to complete digital assessments. A: Sweatband to ensure tight fitting of Dreem 2; B: Dreem 2 electroencephalography (EEG) headband; C: Nonin 3150 WristOx 2 oximeter; D: USB-C charging cable (for Dreem 2 and AX3); E: Axivity AX3 actigraph and wrist strap; F: Saliva collection aid for passive drool; G: Collection tube for passive drool; H: Storage tube for oral swab; I: Oral swab.

Participants were provided with a study kit (Figure 1) consisting of an Axivity AX3 actigraphy watch (Axivity Ltd); Dreem 2 wireless sleep EEG headband (Dreem) [23]; a USB charger; a home saliva collection kit for passive drool (for a dim-light melatonin onset assay); oral swabs (for a cortisol awakening response assay); and an overnight pulse oximeter. Participants who requested a study device were provided with a tablet. Details on devices and the study kit are provided in Multimedia Appendix 2*.* All participants were provided with a printed participant guide with instructions for each remote study task and research team contact details.

At baseline, participants were provided with support to download and use the MyDignio (Dignio AS) app, a telemedicine cloud-based software tool that was used to deliver digital sleep diaries, questionnaires, and reminders to complete study tasks. Participants were also familiarized with the study schedule and tasks and offered support and training ad hoc throughout the study.

#### Main Study Period

Participants completed 56 days of continuous sleep and regular cognitive monitoring in their own home using an Axivity AX3 actigraphy watch and daily sleep diaries [50] delivered via the MyDignio app. Participants were scheduled to complete a set of 3 web-based cognitive tests (choice reaction time, forward digit span, and self-ordered search) twice weekly on a bespoke study version of the web-based assessment platform Cognitron [51].

For 7 days during the 8-week period, participants also completed an “intensive week,” consisting of daily cognitive tasks on Cognitron and nighttime sleep recordings using Dreem 2, a wireless sleep EEG headband. The Dreem sleep staging algorithm has comparable accuracy to manual sleep expert scoring of PSG [23], including in cognitively healthy older adults as well as patients with AD [52] and PD [53]. Sleep recordings were initiated by the participant at their natural bedtime each night and terminated after natural awakening each morning. Data were uploaded via Bluetooth and Wi-Fi to a server accessible to the research team. Additional study tasks during the intensive week included 4 verbal memory assessments involving immediate and delayed free recall and a target-distractor recognition task with a researcher via videoconferencing software and serial saliva samples across 1 evening to assess dim-light melatonin onset and 1 morning to assess cortisol awakening response.

Participants were also invited to undergo 2 nights of pulse oximetry for sleep apnea screening and a blood test for plasma biomarker analysis of Aβ42:40, phosphorylated-tau 181 and 217, neurofilament light chain, and glial fibrillary acidic protein. A protocol amendment approved partway through the study introduced bespoke questionnaires probing study expectations, reasons for participation, experience with technology, and how acceptable they found the intensive week study tasks. Participants were also invited to attend a remote end-of-study interview to share their experiences and asked to return for a 6-month follow-up to complete an MoCA and any outstanding study tasks (eg, missed blood test). Participant feedback from qualitative interviews and questionnaires as well as sleep characteristics for the cohort will be analyzed and presented separately.

### Feasibility and Acceptability Outcomes

#### Recruitment and Retention

Consent, recruitment, and retention rates were recorded and are summarized in a flowchart, alongside reasons for nonparticipation at each stage of the recruitment process.

#### Data Quality and Completeness

For each remote study task, data completeness was assessed by the average number and percentage of completed tasks or nights’ use per participant per participant group (data completeness rate). The number and percentage of individuals who completed the maximum number of data points for a given study task (eg, completed all requested 7 nights of EEG) is also provided. For EEG, signal quality is reported based on Dreem’s automated algorithm. Optimal signal quality is considered ≥85% and good quality is considered ≥70%.

#### Associations Between Participant Characteristics and Adherence

To explore potential associations between key clinical and demographic variables and adherence, we examined the correlation between 2 continuous measures of adherence (number of sleep diaries completed and mean record quality of Dreem EEG data) and 4 continuous variables which might impact digital literacy or engagement (age at consent, apathy, subjective sleep quality as assessed by the PSQI total score, and baseline cognitive impairment as assessed by MoCA total score).

#### Study Support and Resource Use

The number of participants who attended study visits with someone to support them, reported having a partner or relative who could support study tasks, and reported receiving support on at least 1 study task were recorded and are summarized descriptively.

### Data Analysis

Unless otherwise stated, descriptive statistics are provided as mean (SD) for continuous variables, and frequency and percentage for categorical variables, and provided for the full cohort as well as by participant subgroup (AD vs LBD vs controls). Data analysis was performed using R (version 4.3.1; R Foundation for Statistical Computing) and R Studio software (version 2023.6.0.421; R Foundation for Statistical Computing). Actigraphy data were processed using the open-source AX3/AX6 OMGUI application and analyzed in R using the *GGIR* package [54]. Where available, sleep timing was adjusted using participants’ sleep diaries; otherwise, *GGIR* uses the heuristic algorithm looking at distribution of change in z-angle [55] to estimate sleep timing. All files were also visually inspected to check for the accuracy of sleep timing. The quality of the Dreem 2 recordings was assessed by inspecting Dreem’s automated record quality index in the sleep report for each night, which indicates the percentage of the recording that is of scorable quality for sleep analysis. Associations between participant characteristics and adherence were calculated using Spearman rank correlation with α set to *P<*.05. Reasons for nonparticipation and missing data are provided where known and mapped to the Capability, Opportunity, Motivation model of behavior change [56]. Where additional data were collected on any outcome (eg, participants completed an additional night of EEG than instructed), additional data points were removed before analysis to avoid biasing feasibility metrics.

### Patient and Public Involvement

Patient and public involvement (PPI) was sought from individuals with lived experience of MCI and dementia before and throughout the study. PPI contributors reviewed and improved study documents and advised on the acceptability of adding blood biomarker testing. The PPI group strongly endorsed our additional recruitment materials (including an advertisement poster and a plain English participant information sheet) that we introduced partway through the study, after we received feedback from a prospective participant that standard participant information sheets are too long and use inaccessible language for people with cognitive impairment. We also introduced a reduced study protocol, which involved use of a paper (rather than digital) sleep diary, actigraphy, and EEG, to encourage recruitment of participants who may be more comfortable with less frequent use of technology; however, nobody chose the reduced protocol, they either declined altogether or participated in the full study.

## Results

### Participant Characteristics and Enrollment

#### Recruitment

**Figure 2:**
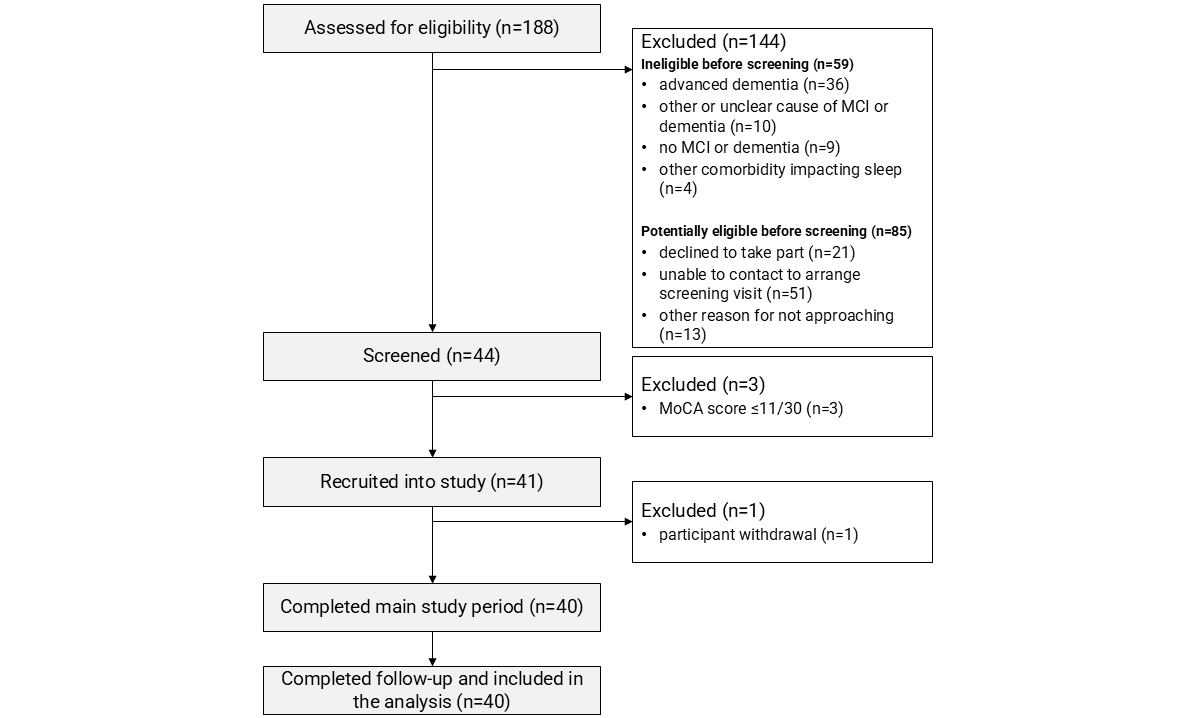
Participant flow through the Remote Evaluation of Sleep to Enhance Understanding of Early Dementia (RESTED) study. MCI: mild cognitive impairment; MoCA: Montreal Cognitive Assessment.

**Figure 3:**
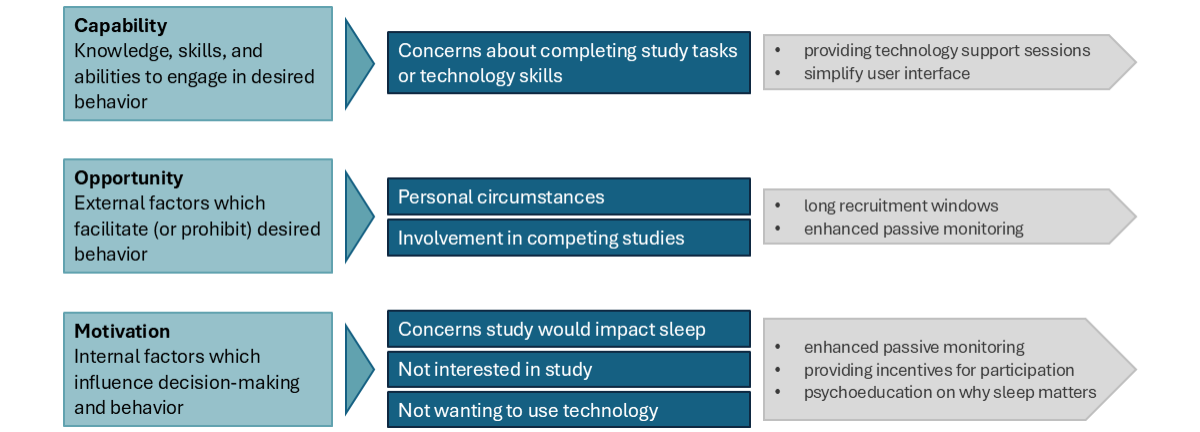
Reasons for declining to participate in the study could be categorized according to the capability, opportunity motivation, behavior (COM-B) model of behavior change in blue. Opportunities to increase capability, opportunity, and motivation to take part in the research are outlined in gray.

Participant flow through the study is shown in Figure 2. Recruitment was open for 17 months from February 2022 to July 2023, with an average recruitment rate of 3 participants per month. Of 129 individuals identified as potentially eligible before screening, 44 individuals consented to take part, giving a consent rate of 34%. Participants described different motivations for taking part, including wanting to support dementia research, perceiving the study as helpful for themselves or others, and because the study sounded interesting or novel. Reasons for declining to take part in the study are described in Figure 3 and mapped to the Capability, Opportunity, Motivation model of behavior change [56].

#### Retention

Of 44 individuals who completed screening, 3 scored less than 11 on the MoCA and were withdrawn. 41 participants were eligible and completed all baseline assessments and training in the at-home study tasks. Of the eligible participants, 1 withdrew before starting the at-home study tasks due to personal circumstances and perceived study burden. All remaining 40 participants completed the main 8-week study period and 6-month follow-up and were included in the analysis, giving a retention rate of 98%.

#### Participant Characteristics

Participant demographics and baseline variables are presented in Table 1. The sample was predominantly made up of male participants with a mean age at consent of 70.9 (SD 5.9, range 57-81) years. Most participants (33/40, 83%) were retired and all identified as White British. In total, 63% (25/40) of participants completed a questionnaire on experiences of digital technology and expectations of the study, which was introduced via an amendment partway through the recruitment period. Of these, most participants reported frequent use of smartphones or other smart technology (18/25, 72%), however only 12% (3/25) of participants had previously used a wearable to monitor their sleep.

**Table 1:** Participant characteristics for the study cohort.

Characteristics			AD^a^ group (n=10)		LBD^b^ group (n=10)		Control group (n=20)
**Diagnosis at consent, n (%)**							
	Mild cognitive impairment	4 (40)		6 (60)		0 (0)	
	Dementia	6 (60)		4 (40)		0 (0)	
**Blood biomarkers of neurodegeneration or AD pathology (pg/mL), mean (SD)**							
	Aβ^c^42:40	0.064 (0.006)		0.062 (0.006)		0.072 (0.007)	
	Phosphorylated-tau 181	26.2 (7.1)		24.9 (12.7)		24.9 (12.7)	
	Phosphorylated-tau 217	0.7 (0.4)		0.8 (0.4)		0.6 (0.4)	
	GFAP^d^	102.2 (28.1)		140.9 (56.4)		103.5 (48.1)	
	NFL^e^	23.9 (18.0)		18.7 (6.5)		13.4 (5.1)	
Age at consent (y), mean (SD)			69.2 (7.9)		73.9 (2.8)		70.3 (5.7)
Female participants, n (%)			2 (20)		2 (20)		5 (25)
**Employment status, n (%)**							
	Full-time employment	2 (20)		0 (0)		2 (10)	
	Part-time employment	0 (0)		0 (0)		3 (15)	
	Retired	8 (80)		10 (100)		15 (75)	
**Education, n (%)**							
	Secondary (≤12 y)	4 (40)		5 (50)		4 (20)	
	Further or higher (>12 y)	6 (60)		5 (50)		16 (80)	
**Comorbid diagnoses,** **n (%)**							
	Obstructive sleep apnea^f^	0 (0)		1 (10)		2 (10)	
	Musculoskeletal or pain	3 (30)		3 (30)		8 (40)	
	Urinary or prostate	3 (30)		2 (20)		3 (15)	
	Depression or anxiety	1 (10)		0 (0)		1 (5)	
**Medications, n (%)**							
	Cognitive enhancer	6 (60)		6 (60)		0 (0)	
	Dopaminergic	0 (0)		8 (80)		0 (0)	
	Melatonin	0 (0)		3 (30)		0 (0)	
	Hypnotic or sedative	0 (0)		2 (20)		1 (5)	
	Antidepressant	4 (40)		4 (40)		4 (20)	
Baseline cognition: MoCA^g^; mean (SD)			23.0 (4.9)		22.0 (4.3)		27.1 (1.4)
Subjective sleep quality: PSQI^h^; mean (SD)			4.3 (2)		7.8 (3)		6.0 (3.9)
Self-reported anxiety: GAD-7^i^; mean (SD)			5.6 (5.2)		3.5 (2.1)		3.6 (4.3)
Self-reported depression: GDS-15^j^; mean (SD)			4.5 (3.3)		4.1 (2.3)		2.2 (2.2)
Self-reported apathy: AES-S^k,l^; mean (SD)			62.2 (6.0)		53.8 (9.9)		62.8 (8.6)

^a^AD: Alzheimer disease.

^b^LBD: Lewy body disease.

^c^Aβ: amyloid beta.

^d^GFAP: glial fibrillary acidic protein.

^e^NFL: neurofilament light chain.

^f^Participants with obstructive sleep apnea were included only if they were being treated with continuous positive airway pressure.

^g^MoCA: Montreal Cognitive Assessment. Higher scores indicate better overall cognition.

^h^PSQI: Pittsburgh Sleep Quality Index. Higher scores indicate worse sleep quality.

^i^GAD-7: Generalized Anxiety Disorder-7. Higher scores indicate more symptoms of anxiety.

^j^GDS-15: Geriatric Depression Scale-15 item scale. Higher scores indicate more symptoms of depression.

^k^AES-S: Apathy Evaluation Scale-Self. Higher scores indicate more apathy.

^l^One participant had a missing value for an item, so this score was imputed using simple imputation.

### Data Quality and Completeness (Adherence)

#### Overview

**Figure 4:**
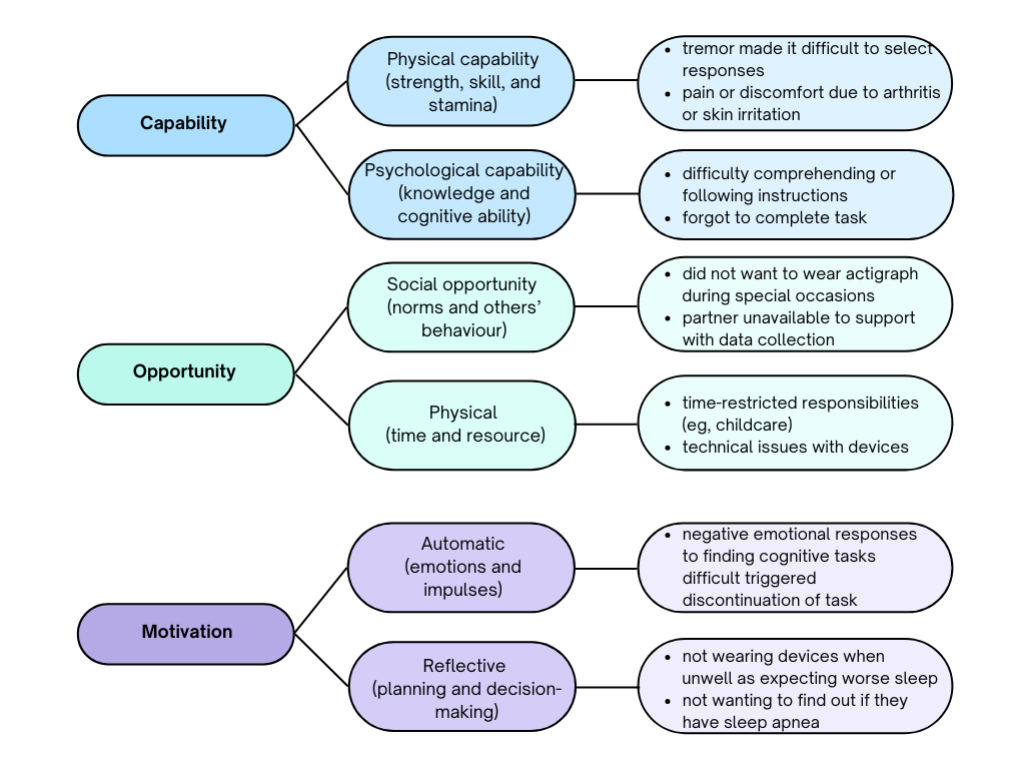
Reasons for missing data are mapped according to the capability, opportunity motivation, behavior (COM-B) model of behavior change.

Adherence to the sleep and cognitive tasks is shown in Table 2. Adherence was very high across all study groups and tasks. Reasons for missing data are summarized in Figure 4 with additional detail provided in Multimedia Appendix 3*.*

**Table 2:** A summary of feasibility outcomes relating to data quality and adherence for wearable devices and remote or web-based cognitive assessments.

Study task (device or software); description; and adherence or data quality metric				AD^a^ (n=10)		LBD^b^ (n=10)		Controls (n=20)		Total (n=40)
**Wearable devices**										
	**Sleep EEG^c^ (Dreem 2): participants were asked to complete a week of consecutive overnight EEG recordings to record nighttime sleep (7 nights)**									
		Nights, mean (SD)	5.9 (2)		6.5 (0.7)		6.6 (0.9)		6.4 (1.2)	
		Full dataset provided (7 nights), n (%)	7 (70)		6 (60)		16 (80)		29 (73)	
		Record quality^d^, mean (SD)	83.5 (12.1)		84.1 (15.0)		85 (18.4)		84.4 (15.8)	
	**Actigraphy (Axivity AX3): participants were asked to complete 24-h wrist actigraphy continuously for 8 weeks (56 nights)**									
		Nights, mean (SD)	50.3 (13.1)		52.2 (6.8)		54.4 (3.2)		52.8 (7.6)	
		Full dataset provided (56 nights), n (%)	5 (50)		5 (50)		14 (70)		24 (60)	
	**Pulse oximetry (Nonin WristOx2 3150): participants were asked to complete 2 consecutive nights of overnight pulse oximetry (2 nights)**									
		Completed ≥1 recording, n (%)	9 (90)		6 (75)		18 (100)		33 (89)	
		Full dataset provided (2 nights), n (%)^e^	6 (60)		5 (63)		15 (83)		26 (70)	
**Remote web-based assessments**										
	**Digital sleep diary (MyDignio): participants were prompted to complete daily sleep diaries for 8 weeks (56 nights)**									
		Diaries completed, mean (SD)	48.2 (13.4)		51.1 (9.8)		53.8 (3.1)		51.7 (8.6)	
		Full dataset provided (56 nights), n (%)	1 (10)		4 (40)		8 (40)		12 (30)	
	**Web-based cognitive tests (Cognitron):** **participants were prompted to complete a digit span, choice reaction time (CRT), and self-ordered search (SOS) tasks on twice weekly for 7 weeks and daily for 1 week (21 tasks)**									
		Digit spans completed, mean (SD)	17.6 (4.3)		17.6 (4.7)		16.8 (4.7)		17.2 (4.5)	
		CRT^f^ completed, mean (SD)	16.9 (4.2)		13.9 (7.5)		16.7 (4.8)		16.1 (5.4)	
		SOS^g^ completed, mean (SD)	17.5 (4.3)		17.5 (4.6)		16.6 (4.7)		17.0 (4.5)	
		Completed >1 web-based cognitive task, n (%)	10 (100)		8 (80)		20 (100)		38 (95)	
		Full dataset provided (all 3 cognitive tasks completed across 21 days), n (%)	2 (20)		2 (20)		2 (10)		6 (15)	
	**Verbal memory tasks (videoconferencing):** **participants were asked to complete 2 evening “learn” tasks and 2 morning recall or recognition tasks scheduled with researchers (4 tasks)**									
		Memory tasks completed, mean (SD)	3.2 (1.7)		3.4 (1.3)		3.9 (0.4)		3.6 (1.1)	
		Full dataset provided (4 tasks completed), n (%)	8 (80)		8 (80)		19 (95)		35 (88)	

^a^AD: Alzheimer disease.

^b^LBD: Lewy body dementia.

^c^EEG: electroencephalography.

^d^Mean first calculated for each participant across all available recordings.

^e^Three participants who already had a diagnosis of sleep apnea were not invited to complete the pulse oximetry.

^f^CRT: Choice Reaction Time.

^g^SOS: Self-ordered search.

#### Sleep EEG

In total, 257 recordings were made using Dreem 2. One participant accidentally completed an eighth recording, which was removed from subsequent analyses to reduce bias.

All participants successfully recorded at least 1 night of sleep, with an average of 6.4 (1.2) nights across the cohort or a 91.4% data completeness rate (Table 2). In total, 73% (29/40) of participants provided data for all 7 nights. Average record quality was also high at 84.4% (15.8), indicating nearly optimal record quality on average across the cohort. One participant wore the Dreem 2 but did not successfully initiate the recording during the intensive week but completed 7 successful nights of recording on a second attempt later in the study. Record quality for individual EEG channels is provided in Multimedia Appendix 4.

#### Actigraphy

In total, 2332 nights of data across the full cohort were collected. A total of 189 of these nights contained no data, either at the beginning or the end of the actigraphy file and were removed from sleep analyses. Following visual inspection, a further 9 nights were removed due to sustained nonwear. Therefore, 2134 nights were of sufficient quality for sleep analysis.

Across the cohort, participants provided an average of 52.8 (7.6) out of 56 analyzable nights, giving a data completeness rate of 94.3%. In total, 60% (24/40) of participants provided 56 analyzable nights. All participants recorded at least 14 days of actigraphy. Unexpected battery failure outside of participants’ control (due to long-term storage without use during the COVID-19 pandemic) affected 2 participants’ recordings and was identified during data check at the midpoint study visit. No participants refused to wear or discontinued use of the actigraphy device, although several reported forgetting or choosing not to wear it on certain days or taking brief breaks due to minor skin irritation.

In total, 37 diary entries were manually entered based on visual analysis of the actigraphy data due to missing sleep diaries and poor heuristic algorithm looking at distribution of change in z-angle algorithm detection of the sleep period. Finally, the sleep analysis software was instructed to solely rely on sleep diary information for 166 nights due to a clear misclassification of sleep period.

#### Overnight Pulse Oximetry

As 3 (8%) out of 40 participants were already being treated for established OSA, 37 (93%) participants were offered overnight pulse oximetry. In total, 3 (8%) participants declined, and 1 (3%) participant was unable to tolerate wearing the device. The remaining 33 (89%) participants completed at least 1 successful overnight recording, with 26 (70%) recording successful oximetry traces on both nights. The data completeness rate was 79.7% for those eligible and asked to complete overnight pulse oximetry. Referrals to a sleep clinic for either sleep apnea or incidental findings (such as abnormal pulse rise index) were indicated for 57% (21/37) of participants. In total, 4 (11%) participants declined referral due to not wanting a formal diagnosis, not wanting to be put on sleep apnea treatment, and the inconvenience of traveling to the clinic. In total, 17 (46%) participants agreed to a referral.

#### Digital Sleep Diaries

Although few participants (12/40, 30%) completed all 56 sleep diaries, overall adherence was high. Participants completed an average of 51.7 (SD 8.6) sleep diaries, and only 2 (17%) participants completed fewer than 75% of their sleep diaries. The data completeness rate was 92.3%.

Only 1 (3%) participant completed sleep diaries on paper while her partner, who facilitated entries into MyDignio, was away. In total, 3 (8%) participants had technical issues with data entry into the MyDignio app lasting several days due to a software update and provided some of their sleep diaries via email which were then entered by the study team.

At the time of study setup, Dignio did not offer data format validation, and free-text response boxes were used for several questions. Participants often did not input data in the format requested, and inconsistency in reporting prohibited reliable automated recoding; however, most were manually interpretable by the research team. Where a range of values were provided, a mean was calculated and the value was rounded to the nearest whole minute (eg, 5-10 min was recoded to 8 min). One diary entry was manually corrected by the research team to align with other diary entries and the actigraphy file. It was not possible to recode some responses due to ambiguity (eg, responses of “not long,” “several minutes,” or “unsure”), leaving 43 completed sleep diaries incomplete on at least one sleep variable. Questions most likely to have a missing value were questions on sleep onset latency and length of nocturnal awakenings.

#### Video-Based Verbal Memory Tasks

In total, 92% (37/40) of participants completed at least 1 verbal memory task with a researcher. Data were missing for 1 (3%) participant as the task was considered inappropriate due to their specific language difficulties, 1 (3%) participant due to technical issues with video calling, and 1 (3%) participant due to distress during the first learning trial. Most participants (35/40, 88%) completed all 4 verbal memory tasks, with 2 (5%) participants completing only 2 of the tasks due to work or childcare commitments. Overall, the data completeness rate was 90%. In total, 3 (8%) participants reported finding the task difficult, 2 (5%) reported feeling distracted during the encoding tasks, 2 (5%) reported finding the encoding task distracting rather than helpful, and 2 (5%) reported finding that usual memory techniques such as the story technique or rehearsing words in an auditory loop were difficult to do in this task.

#### Web-Based Cognitive Tests

Few participants completed all 21 cognitive tasks: 6 (15%) out of 40 participants completed all 3 cognitive tasks on all 21 occasions. In total, 7 (18%) participants provided complete data for choice reaction time tasks, 8 (20%) for the self-ordered search, and 9 (23%) for the digit span task. However, on average data completeness was good, with 79.8% of allocated web-based cognitive tasks completed. Only 1 (3%) participant with PD attempted the tasks but discontinued due to frustration at not being able to do the tasks, as they required speed and accuracy. Several participants anecdotally reported misunderstanding the choice reaction time task and clicking outside of the response window but these instances were not formally recorded.

#### Saliva Samples

##### Overview

The outcomes for saliva and blood samples are summarized in Table 3.

**Table 3:** The outcomes relating to data quality and adherence for biomarker analysis: blood biomarkers, salivary dim-light melatonin, and salivary cortisol awakening response.

Study task (sample type) and description; adherence or data quality metric			AD^a^ (n=10)		LBD^b^ (n=10)		Controls (n=20)		Total (n=40)
**Neurodegenerative and AD biomarker samples (blood):** **participants were asked to have blood drawn for biomarker testing**									
	Participants who provided blood for biomarker testing, n (%)	9 (90)		10 (100)		18 (90)		37 (92)	
	Samples analyzed for all biomarkers, n (%)	8 (80)		8 (80)		14 (70)		30 (75)	
	Samples analyzed for ≥1 biomarker, n (%)	9 (90)		10 (100)		18 (90)		37 (92)	
**Melatonin samples (passive drool):** **participants were asked to complete 7 hourly samples across 1 evening, starting from 5 h before bed, and record the sample time**									
	Analyzable samples provided per participant, mean (SD)	6.3 (2.2)		6.9 (0.3)		7.0 (0)		6.8 (1.1)	
	Participants who completed all 7 samples, n (%)	9 (90)		9 (90)		20 (100)		38 (95)	
	Average discrepancy between scheduled time and reported time of completion (min)^c^, mean (SD)	5.6 (53.4)		−13.9 (32.6)		−9.2 (37.1)		−7.0 (41)	
**Cortisol samples (oral swab):** **participants were asked to complete 3 samples across 1 morning: upon waking up, after 30 min, and after 60 min, and record the sample time**									
	Analyzable samples provided per participant, mean (SD)	2.7 (0.9)		3.0 (0)		3.0 (0)		2.9 (0.5)	
	Participants who completed all 3 samples, n (%)	9 (90)		10 (100)		20 (100)		39(98)	
	Average discrepancy between scheduled time and time completed (min)^c^, mean (SD)	22.5 (27.8)		24.3 (17)		24.6 (23.8)		24.0 (23.2)	

^a^AD: Alzheimer disease.

^b^LBD: Lewy body dementia.

^c^Positive numbers here refer to a sample being taken later than scheduled, and negative numbers refer to a sample being taken earlier than scheduled.

##### Dim-Light Melatonin Assay: Passive Drool Samples

Most saliva samples (272/280, 97.1%) were collected as requested. Of 40 participants, 1 (3%) participant provided no saliva samples, and 1 (3%) participant provided 9 out of 10 samples. Data completeness rate for analyzable samples was 92.5% (259/280) across the cohort. Participant-reported sample times were available for 86.7% (236/272) of the total sample. In total, 50.8% (120/236) of the total sample were reported to have been completed within 15 minutes of the scheduled time, as calculated by average bedtimes reported in the sleep diary. On average, participants completed their samples around 7 minutes early (Table 3). Previous studies have identified highly variable salivary melatonin secretion, with peaks from 2 pg/mL to 84 pg/mL [57]. Across the cohort, values ranged from beneath detectable levels (<1.37 pg/mL) to 92.4 pg/mL.

##### Cortisol Awakening Response: Oral Saliva Swabs

Participants were instructed to provide saliva samples upon waking up, after 30 minutes, and after 60 minutes on one morning during the intensive week. Data completeness was 97.5%, with missing data from only 1 participant who did not provide any saliva samples. Data completeness rate for analyzable samples was 91.7% across the cohort. We compared reported saliva sample timings to their scheduled timings, based on final awakening time estimated by EEG. Participant-reported sample times and EEG awakening time were available for 72.6% (85/117) of cortisol samples. From the 85 samples with timings available, suggested wake times were later than the first sample recording time for 6 samples (7%) and were removed from analysis due to suspected error in recorded saliva timing or date. Participants recorded their first cortisol saliva swab an average of 23 minutes after awakening and overall cortisol samples were generally 24 minutes later than scheduled across the 3 time points (Table 3). Previous studies have identified variable salivary cortisol awakening values from 3 to 19 µg/L in healthy adults [58]. Across the cohort, values ranged from 0.9 to 13.4 µg/L.

##### Associations Between Participant Characteristics and Adherence

There was some evidence of a weak correlation between MoCA score at baseline and EEG record quality, where those with greater cognitive impairment had lower average EEG record quality (r=0.31; *P*=.05). Spearman rank correlations revealed no evidence of correlations between EEG record quality and age (r=−0.10; *P*=.54); apathy (r=0.16; *P*=.31); or PSQI score (r=0.19; *P*=.24). There was also no evidence of correlation between the number of sleep diaries completed and age (r=−0.15; *P*=.35); MoCA score (r=0.11; *P*=.48); apathy (r=0.07; *P*=.69); or PSQI score (r=0.12; *P*=.47).

### Resource Use

#### Support From Partners and Relatives

In total, 36% (4/11) of individuals with AD, 85% (11/13) of individuals with PD or LBD, and 10% (2/20) of controls attended their consent visit with a partner or relative. In total, 80% (32/40) of participants reported that there would be someone external to the research team (eg, partner or relative) who could support them with study tasks if needed at baseline. However, only 30% (12/40) of participants reported receiving support with completing study tasks (n=3, 30% AD; n=8, 80% LBD; and n=1, 5% control). Support from outside of the study team was predominantly from partners and included reminders to complete tasks, setting up devices, and troubleshooting technical problems. Of 40 participants, 3 (8%) participants who required technical support from a partner or relative, or used their devices to complete study tasks, reported missing study tasks due to their partner or relative being unavailable during the study period. The participant who withdrew following baseline reported having someone who could support them at home.

#### Support and Contact With the Research Team

Participants received in person and remote training and support from a researcher to complete study tasks at baseline (including downloading and setting up apps and turning on recording devices) and were offered a refresher training session before the intensive week. Participants were also provided with a written instruction manual, scheduled in-app and email reminders, and ad hoc support as requested by participants or where the team noticed ≥3 consecutive days of missing sleep diary data. All participants had at least 1 in-person visit from a researcher (eg, to download midpoint actigraphy data or retrieve study equipment) during the study period. Participants were advised to contact the research team if they had questions during the study by their preferred method (email, phone, video call, or instant messaging on the MyDignio app). Support from the research team during the study was most often provided by email and involved responding to participant queries on initial setup (eg, downloading the MyDignio app); reminders (eg, tasks to complete or passwords); and technical issues (eg, links not arriving for Cognitron). Some participants also requested telephone-based, video-based, or home-based support (eg, to refresh training on the intensive week study tasks). Most participants did not receive regular reminders from the study team to complete tasks.

#### Device Use

One participant was provided with a study tablet upon request, as they wanted to keep study activities and apps separate to their personal devices. All other participants used their own smart devices for study activities (ie, smartphones, tablets, and personal computers). Most participants used >1 device to complete web-based study tasks due to personal preference or convenience. No devices provided by the study team were lost or damaged during the study, although some participants cut the actigraphy watch strap for comfort.

## Discussion

### Principal Findings

In this study, we show that it is feasible to remotely measure sleep and cognition longitudinally in community-dwelling older adults with MCI and dementia due to AD and LBD and healthy older adults. Eligible participants were interested in, enrolled in, and remained in the study, despite being asked to complete a high volume of remote and novel study tasks across an 8-week period while continuing their usual routines. Only 1 participant withdrew from the study, and this was before remote data collection started. Most participants were receptive to multimodal home-based research using technology and alternatives to in-laboratory sleep assessment. Across the cohort, data completeness rate was high and ranged from 79.8% to 97.1%. Our findings support the use of remote, technology-supported research methods to study natural sleep in future trials and indicate some areas where further improvements and refinements would be helpful. With just under two-thirds of our sample (25/40, 63%) having sleep apnea, and only 3 (8%) being aware of this before joining the study, our results also highlight the importance of sleep apnea screening in older adults, as a risk factor and potentially reversible contributor to cognitive impairment and poor sleep quality [59].

Many older adults with MCI and dementia routinely leverage technology for cognitive stimulation, performing activities of daily living (such as shopping and banking), entertainment, and socializing, and use assistive-technology solutions, such as reminders and navigation to support independence [60]. However, cognitive impairment might impact ability to understand or remember to complete remote or technology-based study tasks, resulting in missing data, and may trigger anxiety if patients feel unable to perform tasks correctly [61]. Although a few previous studies had examined the use of sleep wearables in adults with AD, this was typically done over a brief period, examined feasibility of using only 1 digital health technology, or required input from a carer or study partner [31-34,62]. Our study demonstrated that older adults with MCI and dementia can successfully complete novel multimodal sleep and cognitive assessments, including longitudinal concurrent use of wrist actigraphy, web-based cognitive tests, digital sleep diaries, and wireless EEG headbands. However, more effort will be required to recruit samples which are representative of the older adult population and those with MCI or dementia, as our study predominantly recruited White male individuals who were familiar with smart technology, albeit not the devices used in RESTED.

Data completeness rates, reflecting both the ability to complete study tasks and produce data of analyzable quality, were high across all study tasks and participants. Remote data collection comes with a risk of missing data or nonadherence; however, we were able to mitigate this risk by using devices which upload data to servers at regular intervals (eg, Dreem and Dignio) and, less conveniently, by downloading data manually from devices to check compliance and data quality and offer support where needed (eg, actigraphy). Passive monitoring devices and devices that upload to servers automatically can simultaneously reduce the burden on participants and researchers while minimizing data loss by allowing researchers to monitor and respond quickly to any user or technical issues.

Reasons for missing data were usually known and typically related to infrequent but intentional decisions to remove a device (eg, due to a social event or illness) or, in most cases, technical issues with devices or software beyond the participants’ control, which would not introduce bias. We observed a weak correlation between EEG record quality and baseline cognitive impairment, which could be explained by cognitive impairment impacting ability to correctly wear the EEG headband for maximal impedance or reflect that patients were moving more during the night. Even subtle movements can shift the headband and reduce signal quality and getting optimal signal quality across entire or several recordings is a recognized challenge for dry EEG [52]. More comprehensive cognitive assessment than the MoCA, particularly a deeper assessment of executive dysfunction and long-term memory, might reveal a stronger relationship between cognitive impairment and adherence. However, recent studies using remote monitoring technologies found that, although patients with more advanced neurodegenerative disease or cognitive impairment reported more problems and were less compliant, the study remained feasible [63,64]. Larger studies may also want to consider regression analyses to identify key predictors of adherence to identify where support might be most indicated by the research team or caregiver support. However, importantly, overall record quality remained high across the cohort. Having MCI or mild dementia did not preclude study participation, adherence to remote supervised and unsupervised study tasks, or obtaining good-quality data.

Use of remote sleep and cognitive monitoring can help to partly or fully decentralize research, as participants can use their own devices or be provided with technology via post where needed [65], which is more scalable for clinical trials [66]. Frequent or lengthy clinic visits, particularly those that require overnight stays for sleep analysis, can be burdensome for participants, and may reduce inclusivity by requiring participants to live near a study site or travel long distances. More frequent but briefer remote assessments may also help to mitigate against the risk of large amounts of missing data resulting from a missed clinic appointment, can act as “digital biomarkers” that may detect changes more sensitively than an annual follow-up [67], and are more convenient for participants [68]. Although not the focus of our work, research could also explore the use of digital tools to assess fluctuations in cognition over the day in LBD (where fluctuation in cognition is a key diagnostic criteria) and AD (where a sundowning effect of heightened distress is often observed during the evening hours), both of which are difficult to assess in clinical settings [69,70].

While high adherence has been previously demonstrated in feasibility studies of remote monitoring technologies in participants with cognitive impairment or neurodegenerative conditions, most studies have required intensive study partner support throughout the study period as an eligibility criterion [63,71]. It has been argued that study partners are essential for participant safety and well-being in dementia research, even at the preclinical stage where cognitively normal individuals are enrolled [72]. For some participants, partners or relatives provided essential support for study participation. However, many participants provided good-quality data with minimal to no input from others. Crucially, several participants would not have been able to participate if there had been a study partner requirement. Strict eligibility criteria for trials are a recognized barrier to research participation [73]. Requiring a study partner for all studies may unnecessarily increase the burden for relatives already providing care or support, reducing the participant pool, and may disproportionately affect different groups in society who are underserved by research [74,75]. Our findings encourage researchers to consider inclusive and flexible research designs, including options to formally recruit and collect data from study partners without excluding participants who do not have someone available to support them in research studies.

There are an increasing number of options when considering how to measure sleep and cognition from home, including both research-grade and consumer sleep trackers [76,77]. We opted to use existing technologies that were noninvasive, affordable, required minimal training or supervision, were commercially available research-grade or consumer-grade devices that met necessary data privacy regulations, and provided data in a format that could be analyzed using open-source software. Existing technologies can be implemented immediately, reducing time and cost to setup research studies, have a more mature user interface, and are less error-prone than a newly developed solution, and crucially, may be easier to compare among studies for meta-analysis. Several study tasks required ongoing technical support or services from the manufacturers or developers. As a customer rather than a collaborator, we could not always identify or respond to technical problems, and with some providers, we were not informed in advance of several significant changes, which impacted the study or its participants resulting in data loss. Collaborating with industry, ideally at an early stage of the research process, might ensure longevity and continued support throughout the life course of the study and increased flexibility to adapt technology to better fit research (eg, data validation for digital patient-reported outcomes).

### Limitations

Women, adults aged >80 years, and minoritized ethnic groups were underrepresented in our study, which is often observed in dementia research [78]. Although some barriers (eg, mistrust and accessibility of research) and motivators (eg, altruism) have been identified, further work is needed to identify how to improve research access, inclusion, and participation in dementia research [79]. There may also have been a self-selection bias, whereby individuals who were likely to be more competent and enthusiastic about using technology volunteered to participate in the study. Most of our participants were regular smartphone users, and several prospective participants declined to participate due to concerns regarding their confidence or interest in using the technologies. Although the proportion of older adults in the United Kingdom using the internet is increasing, a significant proportion of older adults do not regularly use the internet or lack fundamental skills such as being able to turn on a device and enter login details [28]. Older adults with MCI and dementia who regularly use devices report that smartphones and tablets can be useful to support activities of daily living, as well as for communication, entertainment, and recreation, but also list concerns, including cybersecurity and vulnerability to fraud [80]. Education around sleep and brain health, and basic digital skills training on how devices can support individuals living with cognitive impairment, may improve perceptions of capability and motivation to participate in similar studies [56,80]. Exploring the feasibility of more passive monitoring technologies, such as mattress sensors or smartphone-based passive sensing, may help to increase participation and confidence from those with less experience or interest in technology. Although these techniques may invite additional concerns around data privacy, passive monitoring technologies are already being used to support older adults aging in place [81,82]. Increasing the pool of prospective participants through recruiting via multiple sites and meaningful engagement with community groups, and considering digital inclusivity is likely to enhance recruitment in future studies. Finally, we did not set a priori feasibility cutoffs for study tasks. Despite these limitations, the high completion and retention rate from the RESTED study suggests that remote sleep and cognitive monitoring could offer detailed sleep profiling suitable for tracking change in sleep over disease progression or for monitoring change in sleep clinical trials in older adults with or at risk of dementia.

### Conclusions

Practical, detailed, and scalable assessment of sleep is essential for understanding how sleep disturbances affect neurodegeneration over time and for developing and evaluating effective interventions. Our results suggest that older adults with MCI and dementia, as well as healthy older adults, can and do engage in multimodal remote sleep and cognitive research, including using wireless EEG, actigraphy, and mobile apps or web applications. Remote monitoring technologies and research designs offer the opportunity to study natural sleep and its relationship with cognition and dementia over extended periods, are scalable, and should be considered when designing future clinical trials in sleep and dementia in these populations.

## Appendix

Multimedia Appendix 1 STROBE (Strengthening the Reporting of Observational Studies in Epidemiology) Statement—checklist of items that should be included in reports of observational studies.

Multimedia Appendix 2 Further information is provided on the devices used to measure sleep and circadian rhythms.

Multimedia Appendix 3 Dreem electroencephalography individual channel metrics.

Multimedia Appendix 4 Additional information on reasons for missing data and the affected study tasks is provided.

## Abbreviations

AD: Alzheimer disease
EEG: electroencephalography
LBD: Lewy body disease
MCI: mild cognitive impairment
MoCA: Montreal Cognitive Assessment
PD: Parkinson disease
PPI: patient and public involvement
PSG: polysomnography
PSQI: Pittsburgh Sleep Quality Index
RESTED: Remote Evaluation of Sleep to Enhance Understanding of Early Dementia
STROBE: Strengthening the Reporting of Observational Studies in Epidemiology

## Data Availability

Data are available from the author upon reasonable request.

## References

[1] Koren T, Fisher E, Webster L, Livingston G, Rapaport P (2023). Prevalence of sleep disturbances in people with dementia living in the community: a systematic review and meta-analysis. Ageing Res Rev.

[2] Postuma RB, Iranzo A, Hu M, Högl B, Boeve BF, Manni R, Oertel WH, Arnulf I, Ferini-Strambi L, Puligheddu M, Antelmi E, Cochen De Cock V, Arnaldi D, Mollenhauer B, Videnovic A, Sonka K, Jung KY, Kunz D, Dauvilliers Y, Provini F, Lewis SJ, Buskova J, Pavlova M, Heidbreder A, Montplaisir JY, Santamaria J, Barber TR, Stefani A, St Louis EK, Terzaghi M, Janzen A, Leu-Semenescu S, Plazzi G, Nobili F, Sixel-Doering F, Dusek P, Bes F, Cortelli P, Ehgoetz Martens K, Gagnon JF, Gaig C, Zucconi M, Trenkwalder C, Gan-Or Z, Lo C, Rolinski M, Mahlknecht P, Holzknecht E, Boeve AR, Teigen LN, Toscano G, Mayer G, Morbelli S, Dawson B, Pelletier A (2019). Risk and predictors of dementia and parkinsonism in idiopathic REM sleep behaviour disorder: a multicentre study. Brain.

[3] Harenbrock J, Holling H, Reid G, Koychev I (2023). A meta-analysis of the relationship between sleep and β-Amyloid biomarkers in Alzheimer’s disease. Biomark Neuropsychiatry.

[4] Zamore Z, Veasey SC (2022). Neural consequences of chronic sleep disruption. Trends Neurosci.

[5] Zielinski MR, Gibbons AJ (2022). Neuroinflammation, sleep, and circadian rhythms. Front Cell Infect Microbiol.

[6] Eshera YM, Gavrilova L, Hughes JW (2024). Sleep is essential for cardiovascular health: an analytic review of the relationship between sleep and cardiovascular mortality. Am J Lifestyle Med.

[7] Seda G, Matwiyoff G, Parrish JS (2021). Effects of obstructive sleep apnea and CPAP on cognitive function. Curr Neurol Neurosci Rep.

[8] Costa YS, Lim AS, Thorpe KE, Colelli DR, Mitchell S, Masellis M, Lam B, Black SE, Boulos MI (2023). Investigating changes in cognition associated with the use of CPAP in cognitive impairment and dementia: a retrospective study. Sleep Med.

[9] Liu WT, Huang HT, Hung HY, Lin SY, Hsu WH, Lee FY, Kuan YC, Lin YT, Hsu CR, Stettler M, Yang C, Wang J, Duh P, Lee K, Wu D, Lee H, Kang J, Lee S, Wong H, Tsai C, Majumdar A (2023). Continuous positive airway pressure reduces plasma neurochemical levels in patients with OSA: a pilot study. Life (Basel).

[10] Sabia S, Fayosse A, Dumurgier J, van Hees VT, Paquet C, Sommerlad A, Kivimäki M, Dugravot A, Singh-Manoux A (2021). Association of sleep duration in middle and old age with incidence of dementia. Nat Commun.

[11] Mayer G, Frohnhofen H, Jokisch M, Hermann DM, Gronewold J (2024). Associations of sleep disorders with all-cause MCI/dementia and different types of dementia - clinical evidence, potential pathomechanisms and treatment options: a narrative review. Front Neurosci.

[12] Blackman J, Swirski M, Clynes J, Harding S, Leng Y, Coulthard E (2021). Pharmacological and non-pharmacological interventions to enhance sleep in mild cognitive impairment and mild Alzheimer's disease: a systematic review. J Sleep Res.

[13] Blackman J, Morrison HD, Lloyd K, Gimson A, Banerjee LV, Green S, Cousins R, Rudd S, Harding S, Coulthard E (2022). The past, present, and future of sleep measurement in mild cognitive impairment and early dementia-towards a core outcome set: a scoping review. Sleep.

[14] Kainec KA, Caccavaro J, Barnes M, Hoff C, Berlin A, Spencer RM (2024). Evaluating accuracy in five commercial sleep-tracking devices compared to research-grade actigraphy and polysomnography. Sensors (Basel).

[15] Tadokoro K, Ohta Y, Hishikawa N, Nomura E, Wakutani Y, Takao Y, Omote Y, Takemoto M, Yamashita T, Abe K (2020). Discrepancy of subjective and objective sleep problems in Alzheimer's disease and mild cognitive impairment detected by a home-based sleep analysis. J Clin Neurosci.

[16] Casagrande M, Forte G, Favieri F, Corbo I (2022). Sleep quality and aging: a systematic review on healthy older people, mild cognitive impairment and Alzheimer’s disease. Int J Environ Res Public Health.

[17] Williams JM, Kay DB, Rowe M, McCrae CS (2013). Sleep discrepancy, sleep complaint, and poor sleep among older adults. J Gerontol B Psychol Sci Soc Sci.

[18] Markun LC, Sampat A (2020). Clinician-focused overview and developments in polysomnography. Curr Sleep Med Rep.

[19] Goparaju B, de Palma G, Bianchi MT Naturalistic sleep tracking in a longitudinal cohort: how long is long enough?. medRxiv.

[20] Blackman J, Morrison H, Lloyd K, Gimson A, Banerjee L, Green S, Cousins R, Rudd S, Harding S, Coulthard E (2022). Sleep measurement heterogeneity in mild cognitive impairment and early dementia - towards a core outcome set: a scoping review. Sleep.

[21] Green SF, Frame T, Banerjee LV, Gimson A, Blackman J, Morrison H, Lloyd K, Rudd S, Frederick Fotherby WG, Bartsch U, Purcell S, Jones M, Coulthard L (2022). A systematic review of the validity of non-invasive sleep-measuring devices in mid-to-late life adults: future utility for Alzheimer's disease research. Sleep Med Rev.

[22] Kourtis LC, Regele OB, Wright JM, Jones GB (2019). Digital biomarkers for Alzheimer's disease: the mobile/ wearable devices opportunity. NPJ Digit Med.

[23] Arnal PJ, Thorey V, Debellemaniere E, Ballard ME, Bou Hernandez A, Guillot A, Jourde H, Harris M, Guillard M, Van Beers P, Chennaoui M, Sauvet F (2020). The Dreem Headband compared to polysomnography for electroencephalographic signal acquisition and sleep staging. Sleep.

[24] Miller DJ, Sargent C, Roach GD (2022). A validation of six wearable devices for estimating sleep, heart rate and heart rate variability in healthy adults. Sensors (Basel).

[25] Cay G, Ravichandran V, Sadhu S, Zisk AH, Salisbury AL, Solanki D, Mankodiya K (2022). Recent advancement in sleep technologies: a literature review on clinical standards, sensors, apps, and AI methods. IEEE Access.

[26] Ding Z, Lee T, Chan AS (2022). Digital cognitive biomarker for mild cognitive impairments and dementia: a systematic review. J Clin Med.

[27] Masanneck L, Gieseler P, Gordon WJ, Meuth SG, Stern AD (2023). Evidence from ClinicalTrials.gov on the growth of digital health technologies in neurology trials. NPJ Digit Med.

[28] Briefing: facts and figures about digital inclusion and older people. Age UK.

[29] Holthe T, Halvorsrud L, Karterud D, Hoel K, Lund A (2018). Usability and acceptability of technology for community-dwelling older adults with mild cognitive impairment and dementia: a systematic literature review. Clin Interv Aging.

[30] Chien SY, Zaslavsky O, Berridge C (2024). Technology usability for people living with dementia: concept analysis. JMIR Aging.

[31] Kent BA, Casciola AA, Carlucci SK, Chen M, Stager S, Mirian MS, Slack P, Valerio J, McKeown MJ, Feldman HH, Nygaard HB (2022). Home EEG sleep assessment shows reduced slow-wave sleep in mild-moderate Alzheimer's disease. Alzheimers Dement (N Y).

[32] Pavlickova H, Russell AE, Lightman S, McCabe R (2021). Feasibility of salivary cortisol collection in patients and companions attending dementia diagnostic meetings in memory clinics. BMC Res Notes.

[33] Jones C, Moyle W (2020). A feasibility study of Dreampad™ on sleep, wandering and agitated behaviors in people living with dementia. Geriatr Nurs.

[34] Guu TW, Brem A, Albertyn CP, Kandangwa P, Aarsland D, Ffytche D (2024). Wrist-worn actigraphy in agitated late-stage dementia patients: a feasibility study on digital inclusion. Alzheimers Dement.

[35] Gabb VG, Blackman J, Morrison HD, Biswas B, Li H, Turner N, Russell GM, Greenwood R, Jolly A, Trender W, Hampshire A, Whone A, Coulthard E (2024). Remote evaluation of sleep and circadian rhythms in older adults with mild cognitive impairment and dementia: protocol for a feasibility and acceptability mixed methods study. JMIR Res Protoc.

[36] von Elm E, Altman DG, Egger M, Pocock SJ, Gøtzsche PC, Vandenbroucke JP (2007). The Strengthening the Reporting of Observational Studies in Epidemiology (STROBE) statement: guidelines for reporting observational studies. Lancet.

[37] McKhann GM, Knopman DS, Chertkow H, Hyman BT, Jack CR, Kawas CH, Klunk WE, Koroshetz WJ, Manly JJ, Mayeux R, Mohs RC, Morris JC, Rossor MN, Scheltens P, Carrillo MC, Thies B, Weintraub S, Phelps CH (2011). The diagnosis of dementia due to Alzheimer's disease: recommendations from the National Institute on Aging-Alzheimer's Association workgroups on diagnostic guidelines for Alzheimer's disease. Alzheimers Dement.

[38] Albert MS, DeKosky ST, Dickson D, Dubois B, Feldman HH, Fox NC, Gamst A, Holtzman DM, Jagust WJ, Petersen RC, Snyder PJ, Carrillo MC, Thies B, Phelps CH (2011). The diagnosis of mild cognitive impairment due to Alzheimer's disease: recommendations from the National Institute on Aging-Alzheimer's Association workgroups on diagnostic guidelines for Alzheimer's disease. Alzheimers Dement.

[39] Yamada M, Komatsu J, Nakamura K, Sakai K, Samuraki-Yokohama M, Nakajima K, Yoshita M (2020). Diagnostic criteria for dementia with Lewy bodies: updates and future directions. J Mov Disord.

[40] Litvan I, Goldman JG, Tröster AI, Schmand BA, Weintraub D, Petersen RC, Mollenhauer B, Adler CH, Marder K, Williams-Gray CH, Aarsland D, Kulisevsky J, Rodriguez-Oroz MC, Burn DJ, Barker RA, Emre M (2012). Diagnostic criteria for mild cognitive impairment in Parkinson's disease: movement disorder society task force guidelines. Mov Disord.

[41] Blackman J, Morrison HD, Gabb V, Biswas B, Li H, Turner N, Jolly A, Trender W, Hampshire A, Whone A, Coulthard E (2023). Remote evaluation of sleep to enhance understanding of early dementia due to Alzheimer's Disease (RESTED-AD): an observational cohort study protocol. BMC Geriatr.

[42] Blackman J, Gabb VG, Carrigan N, Wearn A, Meky S, Selwood J, Desai B, Piggins HD, Turner N, Greenwood R, Coulthard E (2024). Sleep quality during and after severe acute respiratory syndrome coronavirus 2 (COVID-19) lockdowns in the UK: results from the SleepQuest study. J Sleep Res.

[43] Nasreddine ZS, Phillips NA, Bédirian V, Charbonneau S, Whitehead V, Collin I, Cummings JL, Chertkow H (2005). The Montreal Cognitive Assessment, MoCA: a brief screening tool for mild cognitive impairment. J Am Geriatr Soc.

[44] Buysse DJ, Reynolds CF, Monk TH, Berman SR, Kupfer DJ (1989). The Pittsburgh Sleep Quality Index: a new instrument for psychiatric practice and research. Psychiatry Res.

[45] Johns MW (1991). A new method for measuring daytime sleepiness: the Epworth sleepiness scale. Sleep.

[46] Chung F, Abdullah HR, Liao P (2016). STOP-bang questionnaire: a practical approach to screen for obstructive sleep apnea. Chest.

[47] Yesavage JA, Sheikh JI, Brink TL (2008). Geriatric Depression Scale (GDS): recent findings and development of a shorter version. Clinical Gerontology: A Guide to Assessment and Intervention.

[48] Spitzer RL, Kroenke K, Williams JB, Löwe B (2006). A brief measure for assessing generalized anxiety disorder: the GAD-7. Arch Intern Med.

[49] Marin RS, Biedrzycki RC, Firinciogullari S (1991). Reliability and validity of the apathy evaluation scale. Psychiatry Res.

[50] Carney CE, Buysse DJ, Ancoli-Israel S, Edinger JD, Krystal AD, Lichstein KL, Morin CM (2012). The consensus sleep diary: standardizing prospective sleep self-monitoring. Sleep.

[51] Cognitron.

[52] Ravindran KKG, Della Monica C, Atzori G, Nilforooshan R, Hassanin H, Revell V, Dijk DJ (2025). Evaluation of Dreem headband for sleep staging and EEG spectral analysis in people living with Alzheimer's and older adults. Sleep.

[53] González DA, Wang D, Pollet E, Velarde A, Horn S, Coss P, Vaou O, Wang J, Li C, Seshadri S, Miao H, Gonzales MM (2024). Performance of the Dreem 2 EEG headband, relative to polysomnography, for assessing sleep in Parkinson's disease. Sleep Health.

[54] Migueles JH, Rowlands AV, Huber F, Sabia S, van Hees VT (2022). GGIR: a research community–driven open source R package for generating physical activity and sleep outcomes from multi-day raw accelerometer data. J Meas Phys Behav.

[55] van Hees VT, Sabia S, Jones SE, Wood AR, Anderson KN, Kivimäki M, Frayling TM, Pack AI, Bucan M, Trenell MI, Mazzotti DR, Gehrman PR, Singh-Manoux BA, Weedon MN (2018). Estimating sleep parameters using an accelerometer without sleep diary. Sci Rep.

[56] Michie S, van Stralen MM, West R (2011). The behaviour change wheel: a new method for characterising and designing behaviour change interventions. Implement Sci.

[57] Burgess HJ, Fogg LF (2008). Individual differences in the amount and timing of salivary melatonin secretion. PLoS One.

[58] Trilck M, Flitsch J, Lüdecke DK, Jung R, Petersenn S (2005). Salivary cortisol measurement--a reliable method for the diagnosis of Cushing's syndrome. Exp Clin Endocrinol Diabetes.

[59] Naismith S, Winter V, Gotsopoulos H, Hickie I, Cistulli P (2004). Neurobehavioral functioning in obstructive sleep apnea: differential effects of sleep quality, hypoxemia and subjective sleepiness. J Clin Exp Neuropsychol.

[60] Holthe T, Halvorsrud L, Lund A (2022). Digital assistive technology to support everyday living in community-dwelling older adults with mild cognitive impairment and dementia. Clin Interv Aging.

[61] Ahuja M, Siddhpuria S, Reppas-Rindlisbacher C, Wong E, Gormley J, Lee J, Patterson C (2022). Sleep monitoring challenges in patients with neurocognitive disorders: a cross-sectional analysis of missing data from activity trackers. Health Sci Rep.

[62] Van Den Berg JF, Van Rooij FJ, Vos H, Tulen JH, Hofman A, Miedema HM, Neven AK, Tiemeier H (2008). Disagreement between subjective and actigraphic measures of sleep duration in a population-based study of elderly persons. J Sleep Res.

[63] Muurling M, de Boer C, Hinds C, Atreya A, Doherty A, Alepopoulos V, Curcic J, Brem A, Conde P, Kuruppu S, Morató X, Saletti V, Galluzzi S, Vilarino Luis E, Cardoso S, Stukelj T, Kramberger MG, Roik D, Koychev I, Hopøy AC, Schwertner E, Gkioka M, Aarsland D, Visser PJ, RADAR-AD consortium (2024). Feasibility and usability of remote monitoring in Alzheimer's disease. Digit Health.

[64] Maas BR, Speelberg DH, de Vries G, Valenti G, Ejupi A, Bloem BR, Darweesh SK, de Vries NM (2024). Patient experience and feasibility of a remote monitoring system in Parkinson’s disease. Mov Disord Clin Pract.

[65] Aiyegbusi OL, Davies EH, Myles P, Williams T, Frost C, Haroon S, Hughes SE, Wilson R, McMullan C, Subramanian A, Nirantharakumar K, Calvert MJ (2023). Digitally enabled decentralised research: opportunities to improve the efficiency of clinical trials and observational studies. BMJ Evid Based Med.

[66] Inan OT, Tenaerts P, Prindiville SA, Reynolds HR, Dizon DS, Cooper-Arnold K, Turakhia M, Pletcher MJ, Preston KL, Krumholz HM, Marlin BM, Mandl KD, Klasnja P, Spring B, Iturriaga E, Campo R, Desvigne-Nickens P, Rosenberg Y, Steinhubl SR, Califf RM (2020). Digitizing clinical trials. NPJ Digit Med.

[67] Dodge HH, Zhu J, Mattek NC, Austin D, Kornfeld J, Kaye JA (2015). Use of high-frequency in-home monitoring data may reduce sample sizes needed in clinical trials. PLoS One.

[68] Black BS, Taylor HA, Rabins PV, Karlawish J (2016). Study partners perform essential tasks in dementia research and can experience burdens and benefits in this role. Dementia (London).

[69] O'Dowd S, Schumacher J, Burn DJ, Bonanni L, Onofrj M, Thomas A, Taylor JP (2019). Fluctuating cognition in the Lewy body dementias. Brain.

[70] Canevelli M, Valletta M, Trebbastoni A, Sarli G, D'Antonio F, Tariciotti L, de Lena C, Bruno G (2016). Sundowning in dementia: clinical relevance, pathophysiological determinants, and therapeutic approaches. Front Med (Lausanne).

[71] Godkin FE, Turner E, Demnati Y, Vert A, Roberts A, Swartz RH, McLaughlin PM, Weber KS, Thai V, Beyer KB, Cornish B, Abrahao A, Black SE, Masellis M, Zinman L, Beaton D, Binns MA, Chau V, Kwan D, Lim A, Munoz DP, Strother SC, Sunderland KM, Tan B, McIlroy WE, Van Ooteghem K (2022). Feasibility of a continuous, multi-sensor remote health monitoring approach in persons living with neurodegenerative disease. J Neurol.

[72] Grill JD, Karlawish J (2017). Study partners should be required in preclinical Alzheimer's disease trials. Alzheimers Res Ther.

[73] Mitchell AK, Ehrenkranz R, Franzen S, Han SH, Shakur M, McGowan M, Massett HA (2024). Analysis of eligibility criteria in Alzheimer's and related dementias clinical trials. Sci Rep.

[74] Aranda MP, Marquez DX, Gallagher-Thompson D, Pérez A, Rojas JC, Hill CV, Reyes Y, Dilworth-Anderson P, Portacolone E (2023). A call to address structural barriers to Hispanic/Latino representation in clinical trials on Alzheimer's disease and related dementias: a micro-meso-macro perspective. Alzheimers Dement (N Y).

[75] Wise-Brown A, Brangman SA, Henderson JN, Willis-Parker M, Monroe S, Mintzer JE, Grundman M, Smith J, Doody RS, Lin H, Assman B, Rippon GA, Gonzales R, Assunção SS (2024). Promoting diversity in clinical trials: insights from planning the ALUMNI AD study in historically underrepresented US populations with early symptomatic Alzheimer's disease. EClinicalMedicine.

[76] de Zambotti M, Goldstein C, Cook J, Menghini L, Altini M, Cheng P, Robillard R (2024). State of the science and recommendations for using wearable technology in sleep and circadian research. Sleep.

[77] Lee T, Cho Y, Cha KS, Jung J, Cho J, Kim H, Kim D, Hong J, Lee D, Keum M, Kushida CA, Yoon I, Kim J (2023). Accuracy of 11 wearable, nearable, and airable consumer sleep trackers: prospective multicenter validation study. JMIR Mhealth Uhealth.

[78] Banzi R, Camaioni P, Tettamanti M, Bertele' V, Lucca U (2016). Older patients are still under-represented in clinical trials of Alzheimer's disease. Alzheimers Res Ther.

[79] Gilmore-Bykovskyi AL, Jin Y, Gleason C, Flowers-Benton S, Block LM, Dilworth-Anderson P, Barnes LL, Shah MN, Zuelsdorff M (2019). Recruitment and retention of underrepresented populations in Alzheimer's disease research: a systematic review. Alzheimers Dement (N Y).

[80] Wilson SA, Byrne P, Rodgers SE (2024). 'I'd be lost without my smartphone': a qualitative analysis of the use of smartphones and tablets by people living with dementia, mild cognitive impairment, and their caregivers. Aging Ment Health.

[81] Read E, Woolsey C, Donelle L, Weeks L, Chinho N (2023). Passive remote monitoring and aging in place: a scoping review. Can J Aging.

[82] Cornet VP, Holden RJ (2018). Systematic review of smartphone-based passive sensing for health and wellbeing. J Biomed Inform.

