# Supplementary material for: Longitudinal Remote Sleep and Cognitive Research in Older Adults With Mild Cognitive Impairment and Dementia: Prospective Feasibility Cohort Study
Source: JMIR Aging. 2025 May 28;8:e72824. doi: 10.2196/72824 (PMC12159556; doi:10.2196/72824)
Supplement: Multimedia Appendix 1 [file aging_v8i1e72824_app1.docx]

STROBE Statement—checklist of items that should be included in reports of observational studies

|  | | Item No | Recommendation | | Page  No |
| --- | --- | --- | --- | --- | --- |
| **Title and abstract** | | 1 | (*a*) Indicate the study’s design with a commonly used term in the title or the abstract | | 1 |
|  |  |  | (*b*) Provide in the abstract an informative and balanced summary of what was done and what was found | | 3 |
| Introduction | | | | | |
| Background/rationale | | 2 | Explain the scientific background and rationale for the investigation being reported | | 4-6 |
| Objectives | | 3 | State specific objectives, including any prespecified hypotheses | | 6 |
| Methods | | | | | |
| Study design | | 4 | Present key elements of study design early in the paper | | 6 |
| Setting | | 5 | Describe the setting, locations, and relevant dates, including periods of recruitment, exposure, follow-up, and data collection | | 7 |
| Participants | | 6 | (*a*) Give the eligibility criteria, and the sources and methods of selection of participants. Describe methods of follow-up | | 7-8 |
| Variables | | 7 | Clearly define all outcomes, exposures, predictors, potential confounders, and effect modifiers. Give diagnostic criteria, if applicable | | 8-12 |
| Data sources/ measurement | | 8* | For each variable of interest, give sources of data and details of methods of assessment (measurement). Describe comparability of assessment methods if there is more than one group | | 8-12 |
| Bias | | 9 | Describe any efforts to address potential sources of bias | | 12 |
| Study size | | 10 | Explain how the study size was arrived at | | 8 |
| Quantitative variables | | 11 | Explain how quantitative variables were handled in the analyses. If applicable, describe which groupings were chosen and why | | 12 |
| Statistical methods | | 12 | (*a*) Describe all statistical methods, including those used to control for confounding | | 12 |
|  |  |  | (*b*) Describe any methods used to examine subgroups and interactions | | 12 |
|  |  |  | (*c*) Explain how missing data were addressed | | 12 |
|  |  |  | (*d*) *Cohort study*—If applicable, explain how loss to follow-up was addressed | | n/a |
|  |  |  | (*e*) Describe any sensitivity analyses | | n/a |
| Results | | | | | |
| Participants | 13 | (a) Report numbers of individuals at each stage of study—e.g., numbers potentially eligible, examined for eligibility, confirmed eligible, included in the study, completing follow-up, and analysed | | Figure | |
|  |  | (b) Give reasons for non-participation at each stage | | Figure | |
|  |  | (c) Consider use of a flow diagram | | Figure | |
| Descriptive data | 14* | (a) Give characteristics of study participants (eg demographic, clinical, social) and information on exposures and potential confounders | | 14-16 | |
|  |  | (b) Indicate number of participants with missing data for each variable of interest | | 17-19 | |
|  |  | (c) Summarise follow-up time (e.g., average and total amount) | | 15 | |
| Outcome data | 15 | Report numbers of outcome events or summary measures over time | | 13-27 | |
| Main results | 16 | (*a*) Give unadjusted estimates and, if applicable, confounder-adjusted estimates and their precision (eg, 95% confidence interval). Make clear which confounders were adjusted for and why they were included | | 13-27 | |
|  |  | (*b*) Report category boundaries when continuous variables were categorized | | n/a | |
|  |  | (*c*) If relevant, consider translating estimates of relative risk into absolute risk for a meaningful time period | | n/a | |
| Other analyses | 17 | Report other analyses done—e.g., analyses of subgroups and interactions, and sensitivity analyses | | 14-27 | |
| Discussion | | | | | |
| Key results | 18 | Summarise key results with reference to study objectives | | 28-31 | |
| Limitations | 19 | Discuss limitations of the study, taking into account sources of potential bias or imprecision. Discuss both direction and magnitude of any potential bias | | 31-32 | |
| Interpretation | 20 | Give a cautious overall interpretation of results considering objectives, limitations, multiplicity of analyses, results from similar studies, and other relevant evidence | | 32-33 | |
| Generalisability | 21 | Discuss the generalisability (external validity) of the study results | | 31-32 | |
| Other information | | | | | |
| Funding | 22 | Give the source of funding and the role of the funders for the present study and, if applicable, for the original study on which the present article is based | | 33 | |
