# Supplementary material for: Longitudinal Remote Sleep and Cognitive Research in Older Adults With Mild Cognitive Impairment and Dementia: Prospective Feasibility Cohort Study
Source: JMIR Aging. 2025 May 28;8:e72824. doi: 10.2196/72824 (PMC12159556; doi:10.2196/72824)
Supplement: Multimedia Appendix 2 [file aging_v8i1e72824_app2.docx]

Further information is provided on the devices used to measure sleep and circadian rhythms.

| **Home sleep and circadian monitoring study task** | **Device details** | **Schedule for data collection** |
| --- | --- | --- |
| Actigraphy watch | Axivity AX3 with accompanying wristwatch strap | 56 nights, continuous |
| Wireless EEG headband | Dreem 2 dry-electrode EEG headband | 7 nights, intensive week only |
| Saliva swabs (for cortisol) | Salimetrics® Oral Swabs (Cat. 5001.02)  Pre-labelled Swab Storage Tubes (Cat. 5001.05)  Paper diary for timings | 3 samples across 1 morning in the intensive week |
| Passive drool (for melatonin) | SalivaBio Saliva Collection Aids (Cat. 5016.04)  Pre-labelled Cryovial 2mL (Cat. 5004.01)  Paper diary for timings | 7 samples across 1 evening in the intensive week |
| Overnight pulse oximetry | Nonin WristOx 2® Model 3150 pulse oximeter | 2 consecutive nights across the entire study |
| Study tablet (provided if requested) | Apple iPad | n/a |
