# Supplementary material for: Longitudinal Remote Sleep and Cognitive Research in Older Adults With Mild Cognitive Impairment and Dementia: Prospective Feasibility Cohort Study
Source: JMIR Aging. 2025 May 28;8:e72824. doi: 10.2196/72824 (PMC12159556; doi:10.2196/72824)
Supplement: Multimedia Appendix 3 [file aging_v8i1e72824_app3.docx]

Dreem 2 EEG individual channel metrics

|  | F7_O1 | F8_O2 | Fp1_F8 | F8_F7 | F8_O1 | F7_O2 | Fp1_F7 | Fp1_O1 | Fp1_O2 |
| --- | --- | --- | --- | --- | --- | --- | --- | --- | --- |
| AD | 50.21 | 45.23 | 68.40 | 73.24 | 41.27 | 40.86 | 71.56 | 64.60 | 46.13 |
| LBD | 50.89 | 47.69 | 68.05 | 75.79 | 19.68 | 22.67 | 70.78 | 54.36 | 57.14 |
| Control | 72.31 | 64.48 | 83.77 | 89.67 | NA | NA | 84.18 | 64.74 | 57.44 |
| Data from channels F7_O1, F8_O2,Fp1_F8, F8_F7, and Fp1_F7 were calculated for all participants. Participants enrolled earliest in the recruitment period also had channels for F8_O1 and F7_O2, which had the lowest record quality, whilst for later participants, Dreem switched to providing data for channels Fp1_O1 and Fp1_O2 which had higher record quality.  AD = Alzheimer’s disease; LBD = Lewy body disease | | | | | | | | | |
