# Supplementary material for: Longitudinal Remote Sleep and Cognitive Research in Older Adults With Mild Cognitive Impairment and Dementia: Prospective Feasibility Cohort Study
Source: JMIR Aging. 2025 May 28;8:e72824. doi: 10.2196/72824 (PMC12159556; doi:10.2196/72824)
Supplement: Multimedia Appendix 4 [file aging_v8i1e72824_app4.docx]

Additional information on reasons for missing data and the affected study tasks is provided.

| Reason for missing data | Study task(s) affected | Example |
| --- | --- | --- |
| **Cognitive**  Difficulty following instructions for completing a task  Discontinuing a task due to frustration or distress  Forgetting to complete (or if they had already completed) a task | Wireless EEG headband, actigraphy, cognitive tests, verbal memory tasks  Cognitive tests, verbal memory tasks  Verbal memory task, sleep diary, wireless EEG headband | Participant wore EEG headband but did not follow the instructions to initiate the recording  Participant was not able to focus or remember the task during the word list task presentation so asked to stop  Participant took off the actigraphy watch to bathe and forgot to put it back on |
| **Lifestyle / social**  Planning around usual routines  Perception of others at social events | Saliva samples, verbal memory tasks, cognitive tests  Actigraphy | Participant with childcare responsibilities during the evenings was unable to complete evening memory task calls with a researcher  Participant removed the actigraph as they felt self-conscious to wear it at a formal event |
| **Physiological**  Unable to obtain (sufficient) sample from participant  Unrelated physical illness | Saliva samples, blood draw  Wireless EEG headband, blood draw | Participant was unable to produce sufficient saliva due to dry mouth  Participant skipped a night’s recording due to feeling generally unwell |
| **Physical**  Difficulty physically completing a task  Discomfort triggered device removal | Saliva samples, cognitive tests  Pulse oximetry, actigraphy | Participant could not open the tube for the saliva sample  Participants reported itching on wrist underneath actigraph so took a break from wearing it until itching resolved |
| **Technical**  Battery or device failure  Issues accessing website and/or mobile application due to software upgrades  Issues during sample processing  Technical issues unrelated to the study | Actigraphy, pulse oximetry  Cognitive tests, sleep diaries  Saliva samples, plasma biomarkers  Verbal memory tasks | Actigraphy battery failure caused by long-term storage  Software update caused previous data to be stored in the cache and prohibited new data entry  Melatonin level beneath detectable levels for assay  Participant had an unstable internet connection and was unable to get the volume or microphone on their device to work so could not participate in the verbal memory tasks |
